# Setting the context for a complex dental intervention of role substitution in care homes: Initial process evaluation findings

**DOI:** 10.1111/ger.12749

**Published:** 2024-03-27

**Authors:** Annie Hendry, Sarah R. Baker, Gerry McKenna, Georgios Tsakos, Ivor Chestnutt, Craig Smith, Vicki Jones, Ciaran O'Neill, Alison Jenkins, Rachel Evans, Saif Sayeed Syed, Afshan Mirza, Michelle Harvey, Anup Karki, Kirstie Moons, Fiona Sandom, Michael Donaldson, Caroline Lappin, Karen Shepherd, Paul R. Brocklehurst

**Affiliations:** ^1^ Bangor Institute of Health and Medical Research Bangor University Bangor UK; ^2^ Department for Oral Health, Dentistry and Society University of Sheffield Sheffield UK; ^3^ Centre for Public Health Queens University Belfast Belfast UK; ^4^ Department of Epidemiology and Public Health University College London London UK; ^5^ College of Biomedical and Life Sciences Cardiff University Cardiff UK; ^6^ Division of Cardiovascular Sciences University of Manchester Manchester UK; ^7^ Community Dental Services Aneurin Bevan University Health Board Pontypool UK; ^8^ NWORTH Clinical Trials Unit Bangor University Bangor UK; ^9^ Primary Care Division, Public Health Wales Cardiff UK; ^10^ Health Education and Improvement Wales Nantgarw UK; ^11^ Dental Services, Northern Ireland Health and Social Care Board Belfast UK; ^12^ Community Dental Services, South Eastern Trust Belfast UK; ^13^ Patient and Public Involvement UK

**Keywords:** care home residents, complex interventions, implementation, oral health, process evaluation, randomised controlled trials

## Abstract

**Objectives:**

SENIOR (uSing rolE‐substitutioN In care homes to improve oRal health) is a randomised controlled trial designed to determine whether role substitution could improve oral health for this population. A parallel process evaluation was undertaken to understand context. This paper reports on the first phase of the process evaluation.

**Background:**

The oral health and quality‐of‐life of older adults residing in care homes is poorer than those in the community. Oral health care provision is often unavailable and a concern and challenge for managers. The use of Dental Therapists and Dental Nurses rather than dentists could potentially meet these needs.

**Materials and Methods:**

Semi‐structured interviews were conducted with 21 key stakeholders who either worked or had experience of dependent care settings. Questions were theoretically informed by the: Promoting Action on Research Implementation in Health Services (PAHRIS) framework. The focus was on contextual factors that could influence adoption in practice and the pathway‐to‐impact. Interviews were fully transcribed and analysed thematically.

**Results:**

Three themes (receptive context, culture, and leadership) and 11 codes were generated. Data show the complexity of the setting and contextual factors that may work as barriers and facilitators to intervention delivery. Managers are aware of the issues regarding oral health and seek to provide best care, but face many challenges including staff turnover, time pressures, competing needs, access to services, and financial constraints. Dental professionals recognise the need for improvement and view role substitution as a viable alternative to current practice.

**Conclusion:**

Although role substitution could potentially meet the needs of this population, an in‐depth understanding of contextual factors appeared important in understanding intervention delivery and implementation.

## 1 BACKGROUND

The oral health of dependent older adults residing in care homes is becoming increasingly recognised as a significant dental public health issue.^1^ In the United Kingdom, 40% of the 75–84 age group and 33% of the 85+ age group have dental caries, whereas for those that reside in care homes, this figure is substantially worse (73%).^2^ Approximately, half of all care home residents now retain some of their natural teeth.^3^ With increasing levels of polypharmacy, xerostomia is common and diets are often rich in sugars.^4^ This occurs at a time when self‐care commonly deteriorates with increasing levels of dependency and cognitive impairment, leading to the rapid development of dental caries. This can result in pain and deteriorating oral health‐related quality‐of‐life. It can also exacerbate underlying medical conditions.^5^, ^6^, ^7^ Despite this high level of need, dental service provision within the care home environment is poor and a key concern for a range of stakeholders.^8^, ^9^, ^10^ Access to domiciliary services is difficult and complex to deliver and unscheduled care for dental problems is common.^9^ Oral health care providers have an essential role within an interprofessional team in coordinating health professionals within care homes and improving residents' oral health.^6^, ^9^, ^11^, ^12^, ^13^, ^14^

Evidence from the United Kingdom suggests that Dental Therapists (DTs) and Dental Nurses (DNs) could offer an alternative to using dentists to meet this need.^15^ Both DTs and DNs are regulated professions in the United Kingdom and there is increasing evidence of the effectiveness of using DTs, instead of dentists, to identify and manage dental diseases.^16^, ^17^ Equally, there is evidence to support the use of DTs and DNs within a care home environment.^18^ Following an analysis of dental care home survey data in Wales, Monaghan & Morgan concluded ‘a large proportion of need in care homes could be wholly provided by hygienists or therapists’.^15^ However, robust empirical evidence from definitive trials on the use of these professional roles within this setting is currently lacking and led to the design of a complex dental intervention in care homes ‘uSing rolE‐substitutioN In care homes to improve oRal health’ (SENIOR) trial.

SENIOR is a cluster‐randomised controlled trial to determine whether DTs and DNs could improve the oral health of dentate older adults over 65 years of age residing in care homes.^19^ The trial is being run over a 6‐month period in Wales, Northern Ireland and England. In the intervention arm, DTs assess residents at the start and end point of the intervention period, providing routine restorative care, including the placement of fillings and basic periodontal treatment. Any cases requiring extractions or provision of dentures are referred on for care by a dentist. DNs also visit the care homes on a monthly basis to professionally administer fluoride varnish, oversee the use of high‐strength fluoride toothpaste (5000 ppm) and supervise tooth brushing in accordance with evidence‐based guidance.^20^ The visits from the DNs seek to champion oral health and improve the level of day‐to‐day prevention offered by formal carers among care home managers and staff. As highlighted by Brocklehurst et al.,^20^ ‘there is growing support for the use of change agents in implementation processes’, who facilitate the enactment of complex interventions in complex settings. The DT and DN intervention is being compared with ‘treatment as usual’ and the primary outcome measure is a change in the level of dental plaque as a measure of oral cleanliness.

Process evaluations are commonly run alongside definitive trials to understand the contextual factors that may influence the implementation of the intervention.^21^ Process evaluations are particularly important in care home environments given the complexity of the setting.^22^ The recent revision to the UK Medical Research Council (MRC) guidance for the development and evaluation of complex interventions has placed increasing emphasis on the importance of context and an understanding of the circumstances that influence intervention delivery, in order to successfully drive implementation and change.^23^ A well‐planned, theoretically‐informed process evaluation enables researchers to account for context and adapt interventions accordingly which subsequently aids implementation.^24^ This is important, as contextual information is commonly lacking in many trials and systematic reviews – including those in dentistry – presenting a potential barrier to the transferability of findings.^25^, ^26^ The aim of this first phase of the process evaluation was to understand the context in which SENIOR was to be delivered.

## 2 METHODS

### 2.1 Ethical considerations

This study was reviewed and granted full ethical approval by the Bangor University School of Health Sciences Ethics Committee and was granted LREC approval in 2021 (297182; 21/WA/0116).

### 2.2 Theoretical approach

To provide a theoretical framework for the process evaluation, the research team drew on the ‘Promoting Action on Research Implementation in Health Services’ (PARIHS) framework.^27^ PARIHS was developed from Rogers' Diffusion of Innovations explicitly to challenge the pipeline conceptualisation of implementation.^28^, ^29^ PARIHS comprises of three elements (Evidence, Context, Facilitation), which are commonly considered to be critical to any implementation process and is one of the most cited frameworks.^30^, ^31^ The elements and criteria of the PARIHS framework are provided in Table 1 represented a useful structure to inform SENIOR's process evaluation and was used to create a matrix that mapped the relevant stakeholder groups across the different PARIHS criteria to capture and describe the complexity of the setting.^24^ Working alongside SENIOR's Patient Public Involvement group, this was used to create a set of bespoke semi‐structured interview guides for the different stakeholder groups (Appendix 1).^32^ Phase one of the process evaluation focused on the Context element of PARIHS.

**TABLE 1:** PARIHS Framework criteria.

PARIHS elements/sub elements	PARIHS criteria
Context: receptive context	Clearly acknowledged boundaries
	Appropriate and transparent decision‐making processes
	Power and authority processes
	Resources allocated and feedback provided
	Initiative fits with strategic goals and is a key practice/patient issue
	Receptiveness to change
Context: culture	Able to define culture(s) in terms of prevailing values/beliefs
Context: leadership	Transformational leadership
	Role clarity
	Effective teamwork and organisational structures
	Democratic inclusive decision‐making processes
	Enabling/empowering approach to teaching/learning/managing
Facilitation: role	Doing for others/enabling others
Facilitation: skills and attributes	Doing for others/enabling others

### 2.3 Sampling and data collection

The range and number of stakeholders who were interviewed are provided in Table 2. These included managers of the care homes involved in the trial, Consultants in Dental Public Health, Consultants in Special Care Dentistry, dental professionals involved in preventive programmes in care homes and other relevant academics (e.g. Professor of Nutrition, Geriatrician) across the United Kingdom. As is standard practice for qualitative research, where the emphasis is on eliciting information‐rich cases rather than recruiting a representative sample, a purposive sample of participants were identified based on national or local roles.^33^ A study team contacts and a snowballing technique was used to identify further participants. Audio‐taped interviews were conducted and recorded using virtual platforms, given the impact of COVID on face‐to‐face meetings. All participants were provided with the Participant Information Sheet and gave written informed consent prior to interview. Each interview lasted between 30 and 60 min. Data were anonymised, fully transcribed, and analysed thematically by the same researcher (AH).

**TABLE 2:** Participants.

Participant	Role	Region
#1	Trial statistician	Scotland
#2	Consultant in Special Care Dentistry	Wales
#3	Speech and Language therapist	Wales
#4	Consultant in Dental Public Health	SW England
#5	Dental Nurse	SW England
#6	Dental Nurse	Wales
#7	Trainer in Oral Health	SW England
#8	Professor of nutrition	Northern Ireland
#9	Consultant in Special Care Dentistry	SW England
#10	Geriatrician	Northern Ireland
#11	Consultant in Special Care Dentistry	London
#12	Dentist and academic	NE England
#13	Educator in Oral Health	Wales
#14	Professor of Architecture	London
#15	Dental Therapist	Wales
#16	Clinical Fellow CQC	London
#17	Care home manager	Wales
#18	Care home manager	London
#19	Care home manager (Dementia Care)	Wales
# 20	Care home manager	NW England
#21	Care home manager	NW England

### 2.4 Data analysis

A thematic analysis was undertaken using a flexible, interpretive approach to facilitate the identification of themes or patterns within the data set and to relate these to the different elements within PARIHS.^34^ The first phase of the thematic analysis was familiarisation with the data.^35^ The transcripts were then individually coded and mapped across to the PARIHS framework.^35^ The coding structure is provided in Table 3. Representative quotes of each theme are provided in the results. As we sought to explore the factors that underlie the implementation of the intervention as fully as possible, we explicitly focused on the ‘Context’ domain within the PARIHS framework for this paper.

**TABLE 3:** Coding structure.

Themes mapped onto PARIHS ‘Context’	PARIHS criteria	Codes elicited
Receptive context	Clearly acknowledged boundaries (e.g. physical, social, cultural and system)	5: Relationships
	Appropriate and transparent decision‐making processes	3: Responsibility, power and authority
	Power and authority processes	3: Responsibility, power and authority
	Resources allocated and feedback provided	4: Resource allocation
	Initiative fits with strategic goals and is a key practice/patient issue	1: Initiative fit
	Receptiveness to change	2: Receptiveness to change
Culture	Able to define culture(s) in terms of prevailing values/beliefs	6: Prevailing beliefs of stakeholders
	Values individual staff and clients	6: Prevailing beliefs of stakeholders
	Promotes learning organisation	
	Consistency of individuals role/experience to value relationships with others and teamwork	7: Staff turnover, limited time and training gaps
Leadership	Transformational leadership	8: Training and transformational leadership
	Role clarity	9: Role clarity and consistency
	Effective teamwork and organisational structures	10: Organisational structures and access
	Democratic inclusive decision‐making processes	11: Enabling and empowering
	Enabling/empowering approach to teaching/learning/managing	11: Enabling and empowering

## 3 RESULTS

The interviews revealed the complex nature of the care home environment and the barriers and facilitators to the provision of oral health for residents. Overall, there were three themes and 11 codes generated from the process evaluation. All of the themes and codes generated could be related back to the over‐arching sub‐themes in the ‘Context’ element of PARIHS and are presented below. The elements ‘Evidence’ and ‘Facilitation’ will be discussed in a separate paper, as these relate more to the feasibility and implementation of the SENIOR intervention.

### 3.1 Theme 1: Receptive context

#### 3.1.1 Initiative fit

There was a common view among all the participants interviewed that oral health among care home residents is poor and that overall health is often compromised as a result.

I would think it's poor, you know. I mean, from my experience, and what I've actually seen, the oral hygiene is not great, you know. (Consultant, Special Care Dentistry)

While oral care is part of the care plans, it is not always done well, and things may be missed.

There's the difference between putting a toothbrush round very quickly, and documenting you've done mouth care, but not picking up that actually they've got a mouth full of thrush [acute pseudomembranous candidiasis]. (Speech and Language Therapist)

Care home managers reported that many residents have poor oral health when they arrive at the home, making it very difficult for staff to provide care or to improve existing poor oral health.

We had one resident and he hadn't been to a dentist for years. We got him to the dentist and they said he's got 14 teeth that need extracting. That's the worst. (Care Home Manager)

Residents who have neglected their own oral health prior to arrival at the home were also believed to be more resistant to oral care. Managers also explained that many residents are not affiliated with a dentist or have been removed from practice lists due to non‐attendance. Equally, they found it hard to find dentists who would accommodate new and dependent patients and had difficulty making appointments for residents.

But yes, quite a lot of the residents have had huge problems with their teeth and not registered either or have been registered and not been to the dentist for six years or so. And then when I tried to register with a dentist here on XXXX I struggled because I couldn't get them in. (Care Home Manager)

#### 3.1.2 Receptiveness to change

All interview participants stated that using DTs and DNs could be an excellent way of providing oral care for care home residents, given their role and focus on prevention.

A dentist might be your most expensive resource in there, so you probably want to use them for the things that only a dentist can do. You know, the tooth extractions and things like that, don't make them start at the very beginning when there may be so many competing things. (Dentist, Care Quality Commission)

Care home managers argued that residents did not always need a dentist. Access to DTs and DNs would mean that their residents got the care they needed, and it would be helpful for staff to be able to ask questions and gain knowledge from them on how to prevent disease.

They just need a little bit of attention and someone to check if it's really something that they need attention or not. If it's something dental or it's something else. (Care Home Manager)

#### 3.1.3 Responsibility, power and authority

Care home managers explained that oral health care should be part of every resident's care plan and should be undertaken twice a day.

The care plan stipulates to, for their teeth to be brushed twice a day. (Care Home Manager)

They also felt that all staff should be able to recognise oral conditions, notifying managers who would then refer to a dental professional. However, due to the complex nature of the care home environment this is not always possible.

Dental professionals who were working in care home environments thought that it was everyone's responsibility to contribute to oral health care.

Well, our philosophy is that it's everybody's responsibility ….a HCA (Health Care Assistant) delivering drinks and they notice that a patient is wincing in pain, it's their responsibility to be able to flag that up to somebody and get the person seen. (Dental Nurse)

#### 3.1.4 Resource allocation

One care home manager explained that families are often unable or unwilling to fund dental care, and in most cases the care home has to absorb these costs.

And family members don't want to pay for anything that's any extras. So, most of the times, we struggle. Sometimes, if it is really urgent and we know that the residents are suffering as a result, we fund it and then we recharge those invoices out. Oftentimes, the home gets laboured with the debts. (Care Home Manager)

A manager of a dementia care home said that they had a community dentist who was very good but when they were unavailable nobody else would come as they did not want to treat dementia patients.

He was the community dentist. But when he was not well or on holiday, we haven't had any other access to any other, access to any dentist because they wouldn't treat people with dementia. (Care Home Manager)

Local information on the availability of local dental services was often found to be lacking as it is not held by the hospital or the GP and residents may not be affiliated with a dentist.

It is on our pre assessment paperwork [but often], the hospital can't even give you the additional information you'd require. (Care Home Manager)

#### 3.1.5 Relationships

The relationships between carers and residents were considered crucial for understanding resident preferences and being able to deliver personal care. All participants believed that working with the preferences of the resident is key and understanding how and when they like their oral care done is highly beneficial and can have impact upon other needs and overall health.

But what you tend to find is, because people do have preferences, and you're doing personal care. (Care Home Manager)

Relationships were also important between care home managers and their staff.

And then I also do little tests on my staff. I will say to them…and I'll go by the room numbers, room seven, does he have dentures or does he have his own teeth? That's how I know. (Care Home Manager)

Equally, the relationship between the care home staff and incoming health professionals was considered critical.

So, it's just learning different techniques really which we, which when we go into do one‐to‐ones, that we try to teach them. (Dental Nurse)

### 3.2 Theme 2: Culture

#### 3.2.1 Prevailing beliefs of stakeholders

Dental health professionals thought that high sugar food and drinks were a part of care home culture and that families would often bring sweets and biscuits in for residents without considering the implications for oral health.

They have a lot of sweet things, you rarely see patients with water, it tends to be juice, and that's what they're sipping on, and obviously sugary things, and I think relatives visit, and they bring sugary things. (Dental Therapist)

Dental professionals explained that dieticians may prescribe high sugar supplements for residents with low weights or dietary deficiencies, and it may not always be communicated to others that this carries a risk of promoting dental caries.

So I guess a dietitian would for example want patients to have…or residents to have, high sugar or like build up drinks, which are full of sugar, and to perhaps have those several times throughout the day. (Dental Nurse)

There was also a perception among health professionals that the lack of priority for oral care was intertwined with the carers own beliefs about oral care and the ways in which they looked after their own teeth.

Because lot of them sadly don't see their own oral health as that important, they perhaps don't get [to] the dentist themselves, they're not quite sure how to look after their own mouths. (Consultant in Special Care Dentistry)

One care home manager reported that younger carers seemed to prioritise oral health less than older staff.

What I did notice with the younger generation is when I've gone in to check on the personal care side sometimes some of them had forgotten the teeth. (Care Home Manager)

They don't like the fact what they see in the mouth. They're seeing all the plaque, they're seeing the bacteria, you know, and especially with dentures. (Educator, Oral Health)

#### 3.2.2 Staff turnover, limited time and training gaps

High levels of staff turnover and the use of agency staff were reported to be a challenge to oral health provision, due to a lack of consistency in the approach taken to care.

We've done a lot of sessions where we'll train the staff of the care home, but then a lot of them will be bank staff [so] there's not much consistency within each home. (Dental Nurse)

A common theme across the interviews was the pressure of time on care home staff and the opportunity cost of providing oral care.

But when you have someone that has, diabetes care, foot care, eyes, mouth, incontinent, maybe they're doubly incontinent, then you've got a whole load of care needs. And often… …the oral one is the one that doesn't get taken care of. (Consultant and Lecturer, Special Care Dentistry)

Equally, completing additional paperwork was seen as an extra burden.

They're wandering, they're falling, they're toileting, they've got catheters, they've got pads, they've got so much paperwork…anyway, a check list is great on paper, but it's work. (Speech and Language Therapist)

### 3.3 Theme 3: Leadership

#### 3.3.1 Training and transformational leadership

Some participants felt that DTs and DNs may not always feel confident performing clinical tasks out of the clinical environment and therefore may need additional training to work in care homes. DTs and DNs may require mentorship and reassurance that referrals could be made and there would be access to resources.

We train dental therapists and dental nurses and dentists actually, to work in surgeries. When you're working in a care home…. …. it's someone's house. (Consultant and Lecturer, Special Care Dentistry)

#### 3.3.2 Role clarity and consistency

There was some concern from care home managers that without consistency of care the DTs and DNs would not be able to foster relationships with residents and emphasised the need for a person‐centred approach.

Before the domiciliary dentist came out I briefed him on all the residents that he was going to be seeing, about their behaviour, what works, what doesn't work. We did all that and he didn't have any problems. He actually did the happy dance. (Care Home Manager)

#### 3.3.3 Organisational structures and access

Some of the dental professionals in the study had concerns regarding access to homes and explained that repeat visits may be required if they were unable to access the home.

But again, if you're only going to that care home on that day, you might find that you get eight out of ten patients that won't let you near them, and so it might be a case of then having to go back. (Dental Therapist)

However, care home managers did not share this view and stated that access for DTs and DNs would never be a problem as long as they were arranged in advance.

No, there's no problems. No issues at all. There's toilet facilities, everything's in there. They can even have a cup of tea if they want to. (Care Home Manager)

#### 3.3.4 Enabling and empowering

Further to helping DNs and DTs adapt to care home working, care home managers also suggested that they could facilitate dental visits by providing reclining chairs for residents to sit in or well‐lit areas that could be used for oral health visits.

We have got a big atrium which is well lit. The residents can be wheeled into the atrium and they can go into the bedrooms. (Care Home Manager)

## 4 DISCUSSION

The inclusion of a well‐conducted process evaluation alongside an empirical trial appears key to understanding the contextual factors that influenced the acceptability, fidelity and likely pathway‐to‐impact of the intervention.^21^ The data collected highlighted the complexity of the care home context. Care home staff appear to be aware of the importance of oral health but face many challenges in providing oral care. Many residents had significant levels of plaque or decay on arrival at the home. Equally, a lot of residents were no longer able to care for their own teeth and relied on personal care from staff, who in turn, were under considerable pressure to undertake other care duties and are often working in an environment with a high level of staff turnover. This has been found in other studies in the United Kingdom.^36^, ^37^, ^38^ It appears that residents who were not already prioritising their own oral health prior to entry into the dependent setting were also likely to be more resistant to oral care. This can add further pressures to the staff and is exacerbated by cognitive decline or other challenging displays of behaviour by the resident. The view among many care home managers was that many dentists were reluctant to visit care homes.

Relationships and relational working were key themes highlighted by the process evaluation and was also found in previous work which showed that incentivising the right mix of people to be involved in the design of service provision and aligning the goals of the different staff and needs of individual residents was key.^39^ An important element here is the role of ‘intermediaries’, As argued by Goodwin et al. ‘intermediaries have the potential to be effective, particularly in a care home setting and as a tool for promoting better oral health in dependent older people’.^40^ In terms of knowledge transfer, it has been shown that healthcare professionals can help implement care plans, monitor compliance and transfer knowledge to the wider untrained teams in a care home environment.^41^ Equally, aligning patterns of care to the natural ‘rhythm’ of the home was considered important, along with a mutual appreciation of the challenges both NHS and care home staff face each day.^39^ While highlighting the importance of aligning with the care home ‘rhythm’, the interviews did not provide an explicit mention into the value and specific role of the wider oral health team within the NHS in an aged care facility. This may not have been perceived as part of the care home Context, which the specific focus of this study. Other elements of the PARIHS framework not considered here, such as facilitation, may be better suited to provide more insight into these aspects related to the oral health care provision in care homes.

In common with the findings of a 2019 systematic review, lack of knowledge among staff and residents refusing care were barriers to oral health provision. In relation to the former, inclusive care home‐based training was considered key and would include all care home staff working with residents to promote engagement.^39^, ^42^ Equally, ensuring dementia expertise is integral to routine service provision rather than it being seen as a separate service also aligns to findings in previous work^39^ Finance was also considered to be an important constraint, which concurs with a 2019 study of dependent older people living in rest homes which argued that the availability of finance to fund ongoing oral service provision was a factor in a care home environment.^43^ Without referring to the financial role of NHS oral health services, the cost saving element of role substitution was highlighted in the interviews (‘a dentist might be your most expensive resource’) and there was broad support for using DTs and DNs to facilitate oral health care provision.

This initial phase of the process evaluation running alongside a definitive trial of a complex intervention has benefited from using a theoretical framework and using PARIHS ensured the research team were focused on context of the setting. Including a wide range of stakeholders (such as care home managers, academics from different fields with experience around care homes and ageing, health professionals with different roles and expertise of working in care homes) has also facilitated broader understanding and incorporated different viewpoints. As such, this work builds an understanding of the real‐world context in which the SENIOR trial was conducted and how the intervention can be implemented within a care home environment. On the other hand, the detailed focus on the context may have resulted in the interviews not highlighting other important aspects of facilitation that may be primarily tapping on different domains of the framework. Another limitation is that the sample did not include all categories of care home staff; the views of residents or their families were also not included. Future phases of the process evaluation will endeavour to such participants. While this work can provide a framework for similar trials of complex oral health interventions in care homes, direct transferability of the findings to other care homes may be hindered by the idiosyncrasies of the structure and organisation of care homes, and the different nature and characteristics of service supply and provision within primary dental care in other countries.

Overall, the process evaluation highlighted how important the context of the setting is when considering introducing an intervention to improve oral health. Although there is evidence that DTs and DNs could offer a solution to the problems within the care home sector by providing an alternative to dentists, an in‐depth understanding of the contextual factors in this setting appeared important to understand how the intervention could be delivered and implemented.^15^, ^21^

## 5 CONCLUSION

The care home context is varied and complex. An in‐depth understanding of contextual factors is vital for successful oral health care intervention delivery and long‐term implementation. Further phases of the process evaluation will explore intervention delivery with a focus on the Evidence and Facilitation domains of the PARIHS framework.

## AUTHOR CONTRIBUTIONS

All listed authors fulfil the authorship criteria as set out by the International Committee of Medical Journal Editors. All listed authors contributed substantially toward the conception and design of the work (PRB, GT, GM, SRB, IC, CS, VJ, CO'N, AK, KM, FS, MD, CL, KS), the acquisition, analysis and interpretation of data (AH, AJ, MH, AM, SS), drafting the work (AH, PRB, SRB, GT, GM) and all listed authors approved the final version to be published.

## FUNDING INFORMATION

This study/project was funded by the NIHR Health Services and Delivery Research Programme (NIHR128773). The views expressed are those of the author(s) and not necessarily those of the NIHR or the Department of Health and Social Care.

## CONFLICT OF INTEREST STATEMENT

The authors declare no conflicts of interest.

## Data Availability

Data available from the corresponding author upon request.

## APPENDIX 1 TOPIC GUIDE BASED ON THE PARIHS FRAMEWORK

### 1.1

**Table:**

PARIHS elements/Sub elements	PARIHS criteria	Residents	Care‐home staff	Care‐home manager	DTs and DNs	Directors of community dental services	Commissioners
Context: receptive context	Clearly acknowledged boundaries	When was the last time that you saw a dentist? Does a dentist ever come to see you here at the home?		What is currently in place at your home to look after your residents' teeth? Who usually looks after residents' teeth in (workplace)? How does that process work? Who do you think is the best person to help residents look after their teeth?	What is currently in place in (area/practice) to provide oral care for care home residents? Who is responsible for providing oral care for care home residents? Who do you think this should be? What are the challenges to providing oral health care for care‐home residents? Do you work to any particular guidelines or policy to promote oral health in care‐homes?	Why have you decided to take part in SENIOR? How challenging is it delivering an intervention in care‐homes? What are the limitations/opportunities? Do these challenges shape how future interventions should be developed and implemented? Do you work to any particular guidelines or policy to promote oral health in care homes?	What is currently in place in (country/region) to provide oral care for older people living in care homes? Who is responsible in (country/region) for ensuring oral health is provided to people living in care homes? What are the challenges to providing oral health care to care‐home residents? Do you work to any particular guidelines or policy to promote oral health in care homes?
	Appropriate and transparent decision‐making processes	What happens if you had a painful tooth, what would you do?			How do you think you will manage working with DTs/DNs?		
	Power and authority processes				Any problems with Direct Access or legal restrictions on your ability to care for residents of care homes?		
	Resources allocated and feedback provided	Do you have all that you need to keep your mouth and teeth clean?		What are the barriers to looking after resident's teeth? Is there anything that would make it easier or more difficult to manage?	What will you need to care for residents' oral health?		

	Initiative fits with strategic goals and is a key practice/patient issue	How important is keeping your mouth and teeth clean? What could be the problem if you don't?	Can you tell me about your own experiences of helping with looking after residents' teeth (toothbrushing and/or denture care)?	Why have you decided to take part in SENIOR? Could SENIOR produce any unintended effects? How would you design a service to promote the oral health of residents in care homes?	How important are interventions like SENIOR? Why have you decided to take part in SENIOR?	Could SENIOR produce any unintended effects? How would you design a service to promote the oral health of residents in care homes?	Does SENIOR align with your strategic priorities? Do you think the use of skill‐mix is helpful in this setting? Why? Is there any way that SENIOR could be improved?
Receptiveness to change	What do you normally do to keep your mouth and teeth clean? How often would you like to see someone about your mouth and teeth? How important is prevention for you?	How do you feel about looking after your residents' teeth?	How do you think your residents feel about the health of their teeth? Is this important for you at your home?	Do you agree with using “skill‐mix” to care for residents' oral health? Why? Or why not? Is there anything you would change that could make the implementation of SENIOR more possible?		Do you think that interventions like SENIOR could be easily implemented? Why or why not? Are there any barriers to the implementation of interventions like SENIOR? How could an intervention like SENIOR be facilitated at a strategic level? How do you think the SENIOR intervention would work in the long term?
Context: culture	Able to define culture(s) in terms of prevailing values/beliefs	Would you be prepared to see someone who isn't a dentist to look after your teeth?			Can you tell me about your own experiences of providing oral care for care‐home residents?		
Context:leadership	Transformational leadership				How confident do you think you would be in delivering the SENIOR intervention?		
	Role clarity				Why is your role important in the delivery of SENIOR? Any barriers/enablers? Any overlap or gaps between the DTs and DNs that are used in SENIOR?	Thinking about using “skill‐mix” to promote oral health in care homes: how important are issues such as direct access; legal constraints (e.g. prescribing)? Could clinical leadership be a factor (e.g. the influence of the service lead or the ability of DCPs to develop leadership roles)?	
	Effective teamwork and organisational structures			Do you think the SENIOR intervention is manageable for your staff? Any impact on staff workload?	What is your view about the confidence of DCPs in performing clinical tasks in care homes?	What is your view about the confidence of DCPs in performing clinical tasks in care homes?	
	Democratic inclusive decision‐making processes			How do you think your staff feel about looking after your residents' teeth?			
	Enabling/empowering approach to teaching/learning/managing						
Facilitation: role	Doing for others Enabling others			What do you think the main advantages of having regular visits from DNs/DTs are? Any disadvantages?	How do you plan linking with care home staff to promote the oral health of residents?	Is anything key here refacilitating the implementation of SENIOR?	
Facilitation: skills & attributes	Doing for others Enabling others			How do you think DNs/DTs should liaise with yourself and your staff?	Any additional skills or training that you think you'll need?		
